# Possible association of dose rate and the development of late visual toxicity for patients with intracranial tumours treated with pencil beam scanned proton therapy

**DOI:** 10.1186/s13014-024-02464-z

**Published:** 2024-06-17

**Authors:** Arturs Meijers, Juliane Daartz, Antje-Christin Knopf, Michelle van Heerden, Nicola Bizzocchi, Miriam Varela Vazquez, Barbara Bachtiary, Alessia Pica, Helen A Shih, Damien Charles Weber

**Affiliations:** 1 Center for Proton Therapy, Paul Scherrer Institut, Forschungsstrasse 111, Villigen, 5232 Switzerland; 2 Department of Radiation Oncology, Massachusetts General Hospital, Harvard Medical School, Boston, MA USA; 3 Institute for Medical Engineering and Medical Informatics, School of Life Science FHNW, Muttenz, Switzerland; 4 Department of Radiotherapy and Radiation Oncology, Faculty of Medicine, University Hospital Carl Gustav Carus, Technische Universität Dresden, Dresden, Germany; 5 Department of Radiation Oncology, Massachusetts General Hospital, Boston, MA USA; 6 Department of Radiation Oncology, University Hospital of Zürich, Zürich, Switzerland; 7 Department of Radiation Oncology, Inselspital, Bern University Hospital, University of Bern, Bern, Switzerland

**Keywords:** Proton therapy, Dose rate, Visual toxicity, Radiation induced neuropathy

## Abstract

**Background and purpose:**

Rare but severe toxicities of the optic apparatus have been observed after treatment of intracranial tumours with proton therapy. Some adverse events have occurred at unusually low dose levels and are thus difficult to understand considering dose metrics only. When transitioning from double scattering to pencil beam scanning, little consideration was given to increased dose rates observed with the latter delivery paradigm. We explored if dose rate related metrics could provide additional predicting factors for the development of late visual toxicities.

**Materials and methods:**

Radiation-induced intracranial visual pathway lesions were delineated on MRI for all index cases. Voxel-wise maximum dose rate (MDR) was calculated for 2 patients with observed optic nerve toxicities (CTCAE grade 3 and 4), and 6 similar control cases. Additionally, linear energy transfer (LET) related dose enhancing metrics were investigated.

**Results:**

For the index cases, which developed toxicities at low dose levels (mean, 50 Gy_RBE_), some dose was delivered at higher instantaneous dose rates. While optic structures of non-toxicity cases were exposed to dose rates of up to 1 to 3.2 Gy_RBE_/s, the pre-chiasmatic optic nerves of the 2 toxicity cases were exposed to dose rates above 3.7 Gy_RBE_/s. LET-related metrics were not substantially different between the index and non-toxicity cases.

**Conclusions:**

Our observations reveal large variations in instantaneous dose rates experienced by different volumes within our patient cohort, even when considering the same indications and beam arrangement. High dose rate regions are spatially overlapping with the radiation induced toxicity areas in the follow up images. At this point, it is not feasible to establish causality between exposure to high dose rates and the development of late optic apparatus toxicities due to the low incidence of injury.

## Introduction

Proton therapy is a form of radiotherapy, that uses protons as a source of ionizing radiation [1]. Proton therapy was originally introduced through passively scattered delivery techniques (also known as single and double scattering modalities). In passively scattered modes the entire target volume is exposed to the proton beam multiple times per second. In the late 1990´s, the actively scanned proton beam modality was proposed and developed at Paul Scherrer Institut (PSI) [2]. Due to the laterally scattered and longitudinally modulated beam, the instantaneous dose rates in passively scattered modalities are considerably lower compared to those in the actively scanned modalities. Over the last decade, a rapid switch from passively scattered modalities to actively scanned ones has occurred worldwide [3]. In this transition, no extensive consideration was given to the change in instantaneous dose rates during proton irradiation.

Depending on the range of variation, the dose rate (DR) is known to influence radiobiological effectiveness and radiochemical processes [4–6]. The investigation of DR effects in external beam therapy, including proton therapy, is still limited. However, there is evidence suggesting a potential correlation between DR and relative biological effectiveness (RBE) in conventional photon beams [7–10]. In contrast, such effects have not been identified in carbon ion beams. Noteworthy, very limited data is available on DR effects in proton beams.

Rare, unexpected severe toxicities can occur in patients following proton therapy [11, 12]. A dedicated workshop on these remarkable toxicities was organized by the European Particle Therapy Network in 2022 (https://www.estro.org/Science/EPTN). Some of these reported toxicities occur at relatively low dose levels compared to the dose constraints applied in conventional photon therapy. Therefore, such events are rather unexpected and difficult to explain with a dose-metric paradigm. At PSI, we have observed rare but high-grade visual toxicity in elderly patients with or without hypertension and diabetes [12].

With regards to RBE of proton therapy, a constant factor of 1.1 is generally applied in clinical practice. However, evidence is mounting that RBE along the proton track is variable [13]. In most variable RBE models dose-averaged LET (LET_d_), cell type and fractionation are considered. The available literature provides conflicting evidence regarding the relationship between elevated LET_d_ distributions and the occurrence of late toxicities.

It is our speculation that DR, along with other factors, may contribute to variations in relative biological effectiveness. In this preliminary study, we explore DR related metrics in an attempt to define additional parameters that may be predictive of optic apparatus toxicities. Such investigation was previously suggested by Daartz et al. [14].

## Materials and methods

A selected number of treatment plans for patients with and without visual toxicity was analysed retrospectively. Toxicities to the intracranial visual pathways of these patients were identified during weekly follow-up meetings organized by the Study and Research Office within our department. Radiation induced optic neuropathy (RION) regions were identified on follow up MRI scans as a T1 contrast-enhancement of pre-chiasmatic optic nerve segments and contoured by a radiation oncologist (MD), in the absence of tumour progression. Matched control cases were chosen to represent a similar indication, within a similar tumour location treated at the same time period in the proximity of the optic apparatus as for the index cases with visual toxicities. No case with a follow-up period less than 10 months was considered in the evaluation. As reported in literature the expected development of optic toxicity post-irradiation is 10 to 20 months [15]. The characteristics of the selected cases are shown in Table 1.

**Table 1:** Summary of patient characteristics. Cases are labelled using nomenclature Tx-yy, where Tx indicates the grade of developed visual toxicity and yy indicates the number in the group

Patient	Diagnosis	I/C*	Sex	Age	Pr. dose**, Gy_RBE_	Fx dose, Gy_RBE_	Toxicity grade
T0-01	Meningioma, WHO Grade 2	C	F	47	55.8	1.8	0
T0-02	Meningioma, WHO Grade 1	C	F	59	50.4	1.8	0
T4-03	Mature Teratoma	I	M	34	50.4	1.8	4
T0-04	Meningioma, unspecified	C	F	47	50.4	1.8	0
T0-05	Meningioma, WHO Grade 1	C	F	66	54	1.93	0
T3-06	Meningioma, unspecified	I	F	62	50.4	1.8	3
T0-07	Meningioma, WHO Grade 1	C	M	60	50.4	1.8	0
T0-08	Meningioma, WHO Grade 1	C	M	73	50.4	1.8	0

*Abbreviations* * C: control case; I: Index case ** Prescribed dose

Treatment plans were recalculated using an open-source Monte Carlo (MC) dose calculation engine MCsquare [16]. Every spot in the plan was calculated individually to obtain a set of spot-wise dose distributions. Each spot was simulated with 10^5^ particles. Dose was defined as dose to water. From MC calculations we also retrieved dose-averaged linear energy transfer (LET_d_) distributions, in order to investigate if optic apparatus structures have been located in an elevated LET_d_ region. In the LET_d_ calculation secondary electrons, secondary protons, deuterons, and alphas are taken into account. For each voxel a dose weighted average of the energy loss divided by length travelled by particles is scored [17]. As the highest LET areas may appear at a considerable distance from the primary treatment volume in areas, where little dose is delivered, additionally RBE dose distributions according to McNamara variable RBE model [18], were calculated. For optic apparatus structures α/β ratio of 2 was considered.

Each of the analysed treatment plans was delivered on a ProBeam Connect (Varian Medical systems, Palo Alto, USA) proton therapy system in a dry run, to obtain treatment delivery log files. The log files were used to obtain the delivery time of a spot. The readouts in the log files are affected by uncertainty. The only information obtained from the log files in this study was spot duration. Due to the nature of the pencil beam scanning technique, measuring time structure on the spot-by-spot level is unpractical, therefore we rely on the delivery log files. Spot timing in the log files is recorded with a time resolution of up to 1 µs. The typical spot duration is in the order of several ms. Spot duration in a particular delivery can be affected by day-to-day variations in the setup of the delivery equipment (e.g., cyclotron). Furthermore, interlocks during a delivery of the field can introduce variations in spot durations after resumption of the field. Such scenarios were not considered in the current study, as all spot-wise dose rates were calculated based on a log file from a single uninterrupted dry-run delivery. 3 fields were delivered multiple times to obtain insights in reproducibility of spot durations in uninterrupted deliveries. Variation is spot duration was found to be 0.1 ± 0.9%.

The dose rate per voxel for every spot in the plan was defined as a ratio between dose in the voxel and the delivery time of the spot. The dose and dose rate distributions per spot were used to define and explore various dose and dose rate-related metrics, with a focus on the maximum voxel-wise dose rate (MDR) metric.

MDR is defined on the treatment plan basis and is calculated as the maximum dose rate as seen by a voxel considering all dose rate contributions to the individual spots.

Once the MDR distribution for a plan is defined, based on this distribution, near-maximum (MDR2) dose rates were calculated for the structures which are involved in the optic apparatus: optic chiasm (OC), optic nerve left (ONL) and right (ONR). MDR2 can be considered analogous to the DVH metric D2. MDR2 is near-maximum MDR delivered to 2% of the volume of the structure-of-interest.

## Results

**Fig. 1:**
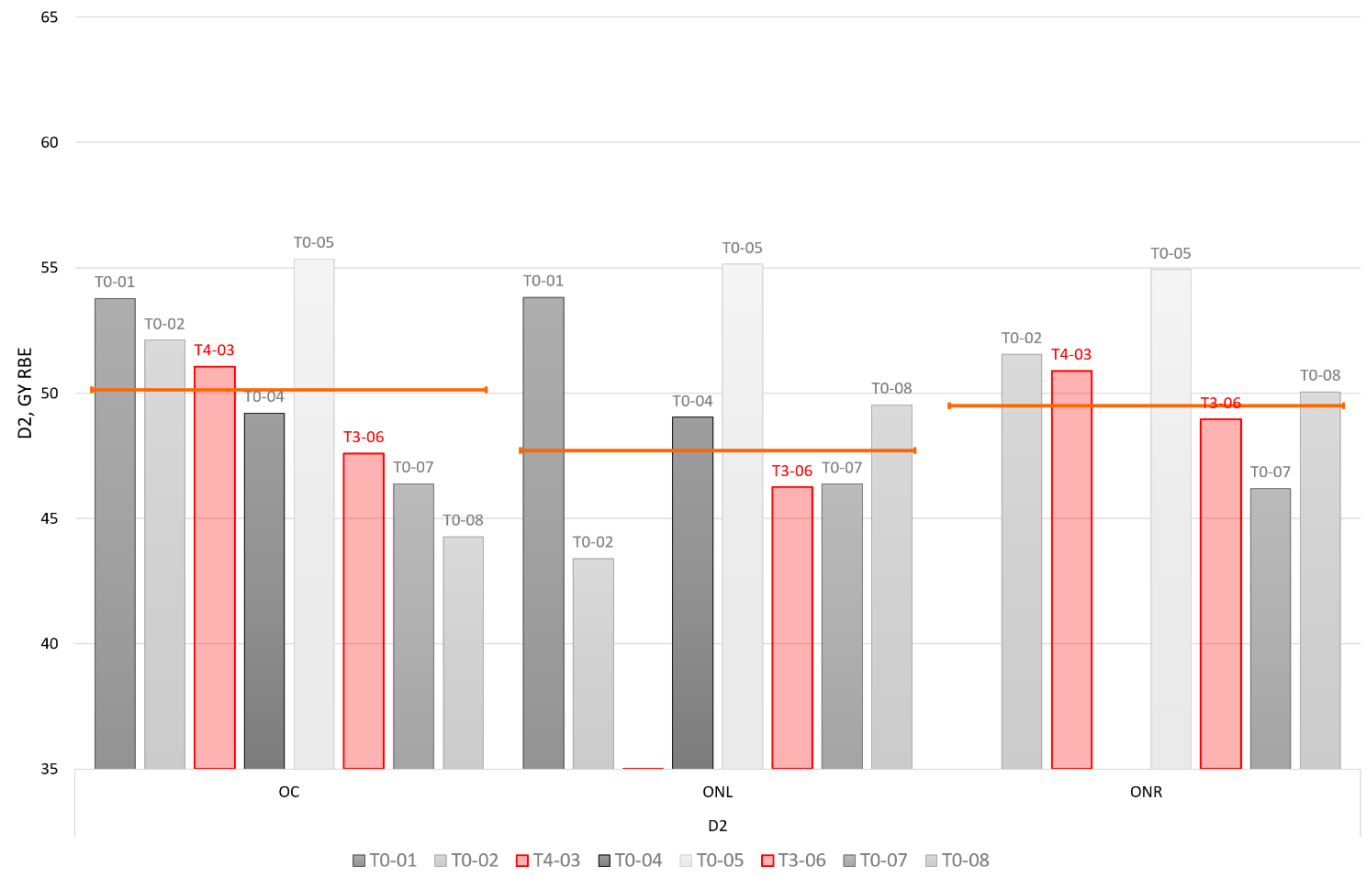
D2 metrics of the optic apparatus structures for the studied patient group. Toxicity index cases are indicated in red. Orange line indicates median D2 value per each considered structure: optic chiasm (OC), optic nerve left and right (ONL, ONR)

Figure 1 demonstrates D2 delivered to the associated structures of the optical apparatus: optic chiasm (OC), left and right optic nerve (ONL, ONR). The optical apparatus of the two toxicity index cases (T4-03 and T3-06) was not exposed to an unusually high dose level (D2) compared to other similar intracranial cases.

Therefore, a toxicity explanation linked solely to dose-volume effects seems unlikely for these two index cases. For cases T4-03 and T3-06 complications developed in the left pre-chiasmatic optic nerve. In both cases, the section with RION, as visible on the follow up MRI, was part of the structure “optic chiasm” in the planning structure set (in accordance with the contouring guidelines).

**Fig. 2:**
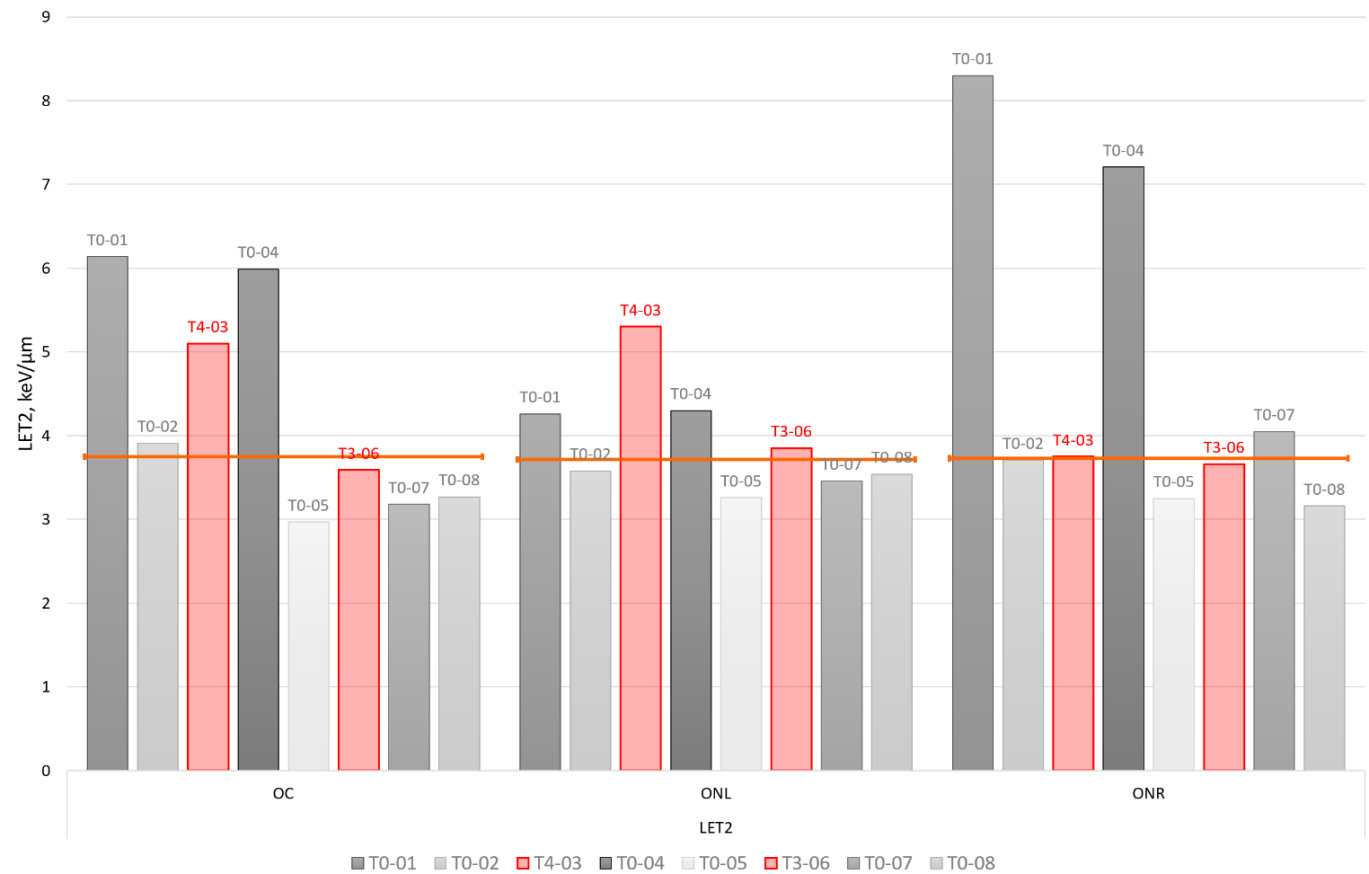
shows near-maximum LET values for the 2 index and 6 control cases analysed in this study. Near-maximum LET is determined as LET delivered to 2% of the volume of the OAR

Figure 2. LET2 metrics of the optical apparatus structures for the studied patient group. Toxicity index cases are indicated in red. Orange line indicates median LET2 value per each considered structure: optic chiasm (OC), optic nerve left and right (ONL, ONR).

The plot shows the highest LET2 for the three structures of the optic apparatus. Similarly, as with dose metrics D2, the toxicity cases do not show unusually high near-maximum LET values in the structures of the optical apparatus. RION, as identified on the follow up MRI, was overlapping with the structure “optic chiasm” in the planning structure set.

**Fig. 3:**
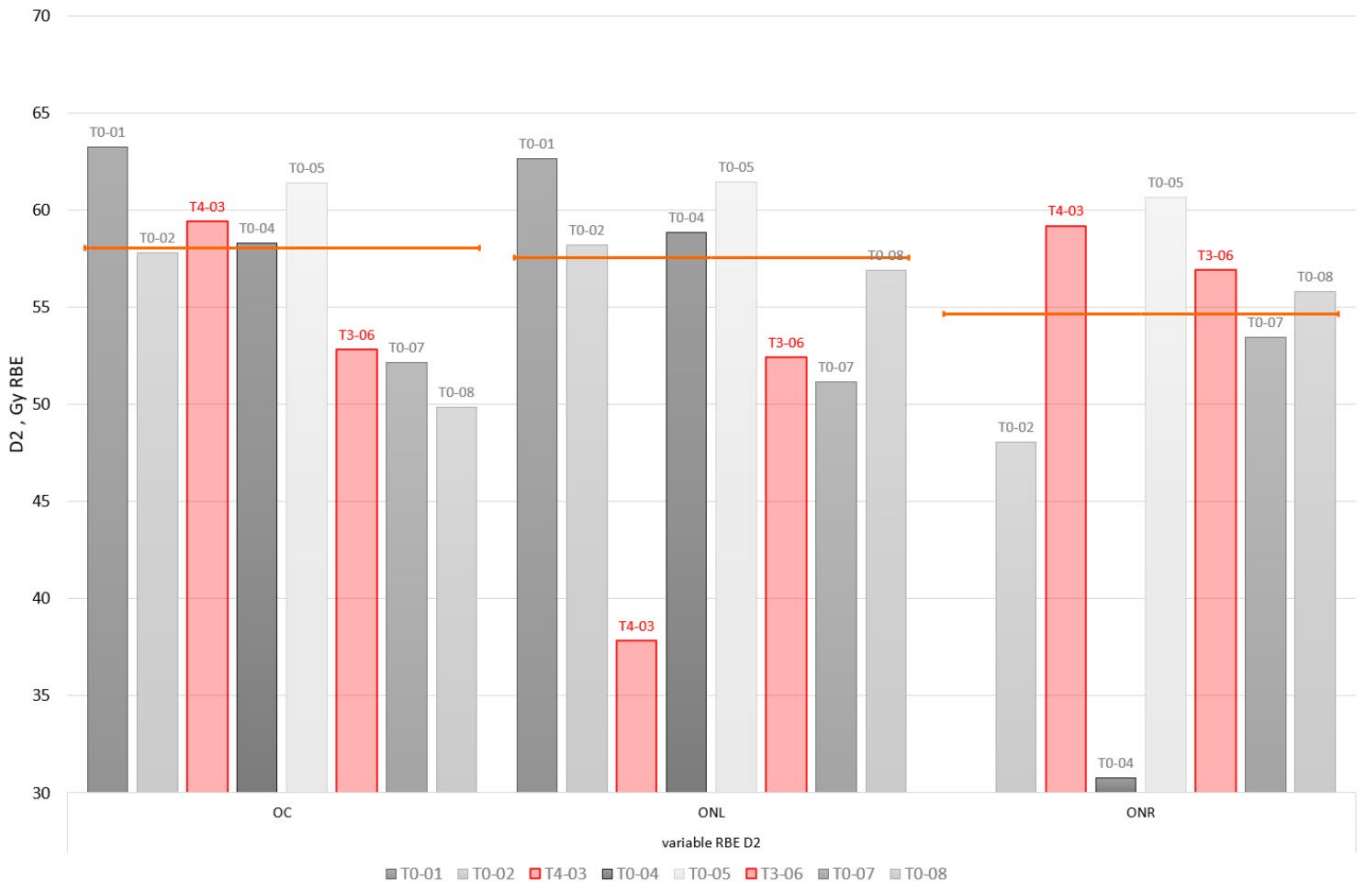
D2 metrics (as per variable RBE model by McNamara) of the optic apparatus structures for the studied patient group. Toxicity index cases are indicated in red. Orange line indicates median D2 value per each considered structure: optic chiasm (OC), optic nerve left and right (ONL, ONR)

Figure 3 shows D2 metrics based on dose distributions as calculated using variable RBE model proposed by McNamara. Although the dose values should be interpreted with caution, since variable RBE models impose considerable uncertainty due to lack of in vivo validation, the approach allows for relative comparison of various treatment plans and their dose contribution to organs at risk.

**Fig. 4:**
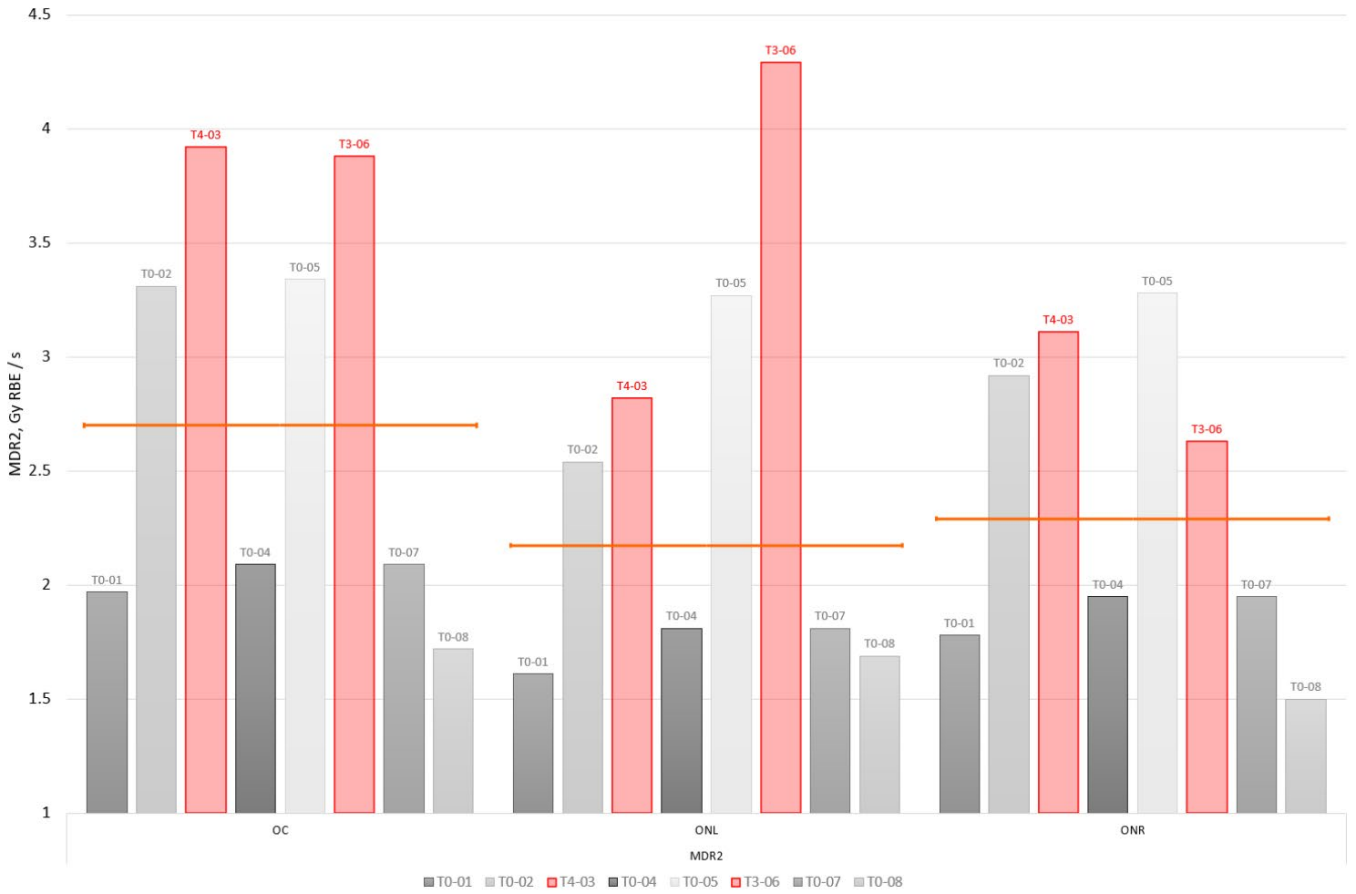
Voxel wise maximum dose rate metrics (MDR2) of the optic apparatus structures for the analysed patient group. Toxicity index cases are indicated in red. Orange line indicates median MDR2 value per each considered structure: optic chiasm (OC), optic nerve left and right (ONL, ONR)

Voxel-wise maximum dose rates (MDR) in the optical apparatus of the 2 index and 6 control cases are shown in Fig. 4. Similarly, as before, the plot shows the maximum MDR as seen by 2% of the volume of any of the three structures in the optical apparatus: OC, ONL, ONR.

**Fig. 5:**
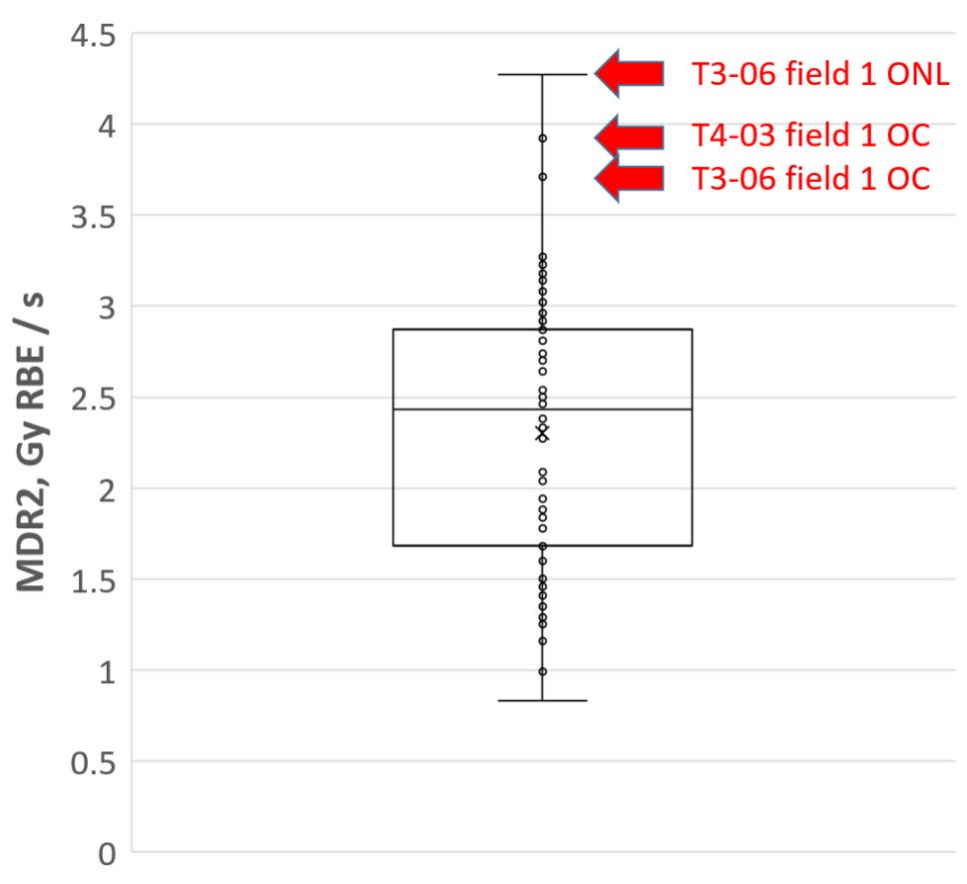
Box plot of field-wise voxel-wise maximum dose rates (MDR2) to any of the optic apparatus structures: optic chiasm (OC), left and right optic nerves (ONL and ONR). Toxicity index cases, which remain unexplained by dose metrics D2, are indicated in red. MDR2 to OC and ONL for the two index cases correspond to the highest values in the dataset

The optic chiasm of the two toxicity cases (T4-03 and T3-06), which could not be explained by high D2 values, appears to be exposed to a higher MDR2 than the optic chiasm of the non-toxicity cases. The high MDR2 values observed in both cases can be attributed to a single field from the treatment plans. As a result, we investigated whether the delivery of such high MDR2 values from a field to an optical structure is unusual within our dataset. Figure 5 shows a box plot of near-maximum MDR2 per field per optical structure. The box plot is constructed of 84 data points, which corresponds to 28 fields (3 to 4 fields per plan) × 3 OARs (OC, ONL, ONR).

The highest data points are associated with the two unexplained toxicity cases: T3-06 and T4-03. This suggests that the exposure of the optical structures to such high dose rates is atypical.

**Fig. 6:**
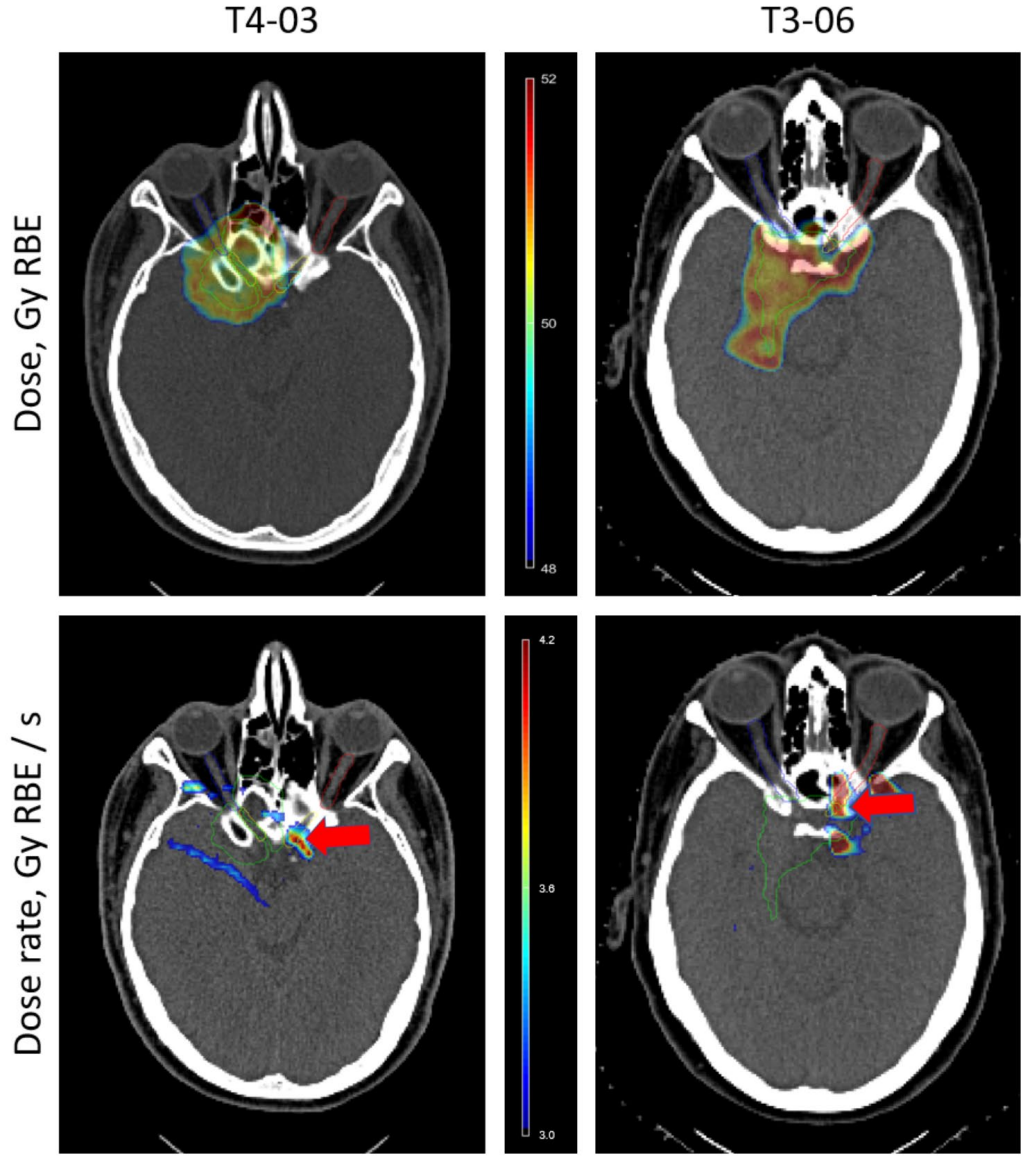
Overlay of dose and voxel wise maximum dose rate (MDR) distributions on CT images of the two toxicity cases (T4-03 and T3-06). Dose rate distributions are shown for the fields contributing the highest MDR values. The maximum dose rate distribution above 3 Gy_RBE_/s threshold is shown. High dose rate areas overlap with pre-chiasmatic left optic nerve areas, which developed radiation induced optic neuropathy (RION) on the follow-up MR images. The optic chiasm is shown in yellow, left and right optic nerves are shown in red and blue, respectively, CTV is shown in green, and area of RION is indicated with a red arrow. The dose and dose-rate colourwashes are displayed at the centre of the Figure

Figure 6 demonstrates MDR maps of the two fields delivering relatively high dose rates to the OC and ONL (case T3-06) and OC (T4-03) overlayed on the planning CTs of the patients

The area, where the high dose rate cloud is overlapping with the structures of the optical apparatus, correlates well with the area of the developed toxicity, as could be identified on MR images.

## Discussion

The analysed patient group consists of 2 index toxicity and 6 similar non-toxicity control cases. The non-toxicity cases were matched by the proximity of the target volume to optical structures as well as the indication for treatment. The patients were similarly treated on PSI Gantry 3 (ProBeam Connect) in a time period between October 2019 and February 2022.

A dose metric D2 above a threshold dose (above 60 Gy_RBE_) [19] increases the risk for development of late optic pathway toxicities. However, it can also be seen that the two toxicity index cases were associated with considerably lower D2 dose levels: 51.05 Gy_RBE_ and 48.95 Gy_RBE_ for the cases T4-03 and T3-06, respectively (Fig. 1). Although significant variability in terms of sensitivity to dose exists in the population, our data suggests the existence of other dose promoting adverse effects. LET is widely considered to be an adverse effect promotor, where essentially elevated LET results in an elevated RBE [20]. LET distributions for the given patient group were calculated. However, similarly as with the dose metrics D2, we did not observe a correlation between elevated LET and the location of radiographic injury in the optic apparatus for the index cases, or a variance in LET between index and control cases to suggest an association with optic apparatus injury (Fig. 2). LET on its own is a parameter that is difficult to interpret in terms of association with increased clinical toxicity risk [21–23]. Numerous studies have demonstrated a lack of association between LET and radiation-induced toxicity while other series have suggested a causal relation [24].

Dose rate in proton pencil beam scanning is a parameter that can be considered from various perspectives: instantaneous, averaged over the duration of the delivery of a field or averaged over a predefined period of time (for example, 100 ms) [14]. Furthermore, when calculating dose rate, the considered dose level can be factored in (for example, dose rate is considered only for delivered dose in the voxel above 50 cGy level). We derived a global voxel wise maximum dose rate as a parameter (MDR) to gain initial insights into the dose rate related parameters in the context of the development of late toxicities. MDR is related to a maximum instantaneous dose rate that a voxel has been exposed to disregarding the amount of dose delivered at this dose rate. Nevertheless, we observed that for the two toxicity cases T4-03 and T3-06, which were difficult to explain in the context of dose metrics, MDR was shown to be elevated compared to other cases in the group (Fig. 4). In fact, MDR2 as high as 3.9 Gy_RBE_/s and 4.3 Gy_RBE_/s was observed only for the two unexplained toxicity index cases (T4-03 and T3-06). These high MDR values could be traced back to a contribution from a single field in the plan for both cases. Figure 5 has shown that such high dose rates delivered from a field to structures of the optical apparatus are atypical.

In this study we demonstrate the relative differences in MDR between index and control cases. A word of caution should be mentioned with regards to broader adoption of absolute scale. The MDR metric is sensitive to dose threshold value, which in the current study was set to 1 cGy per fraction. By adopting different parametrization, the obtained absolute MDR values may vary considerably. The choice of the dose threshold value in MDR calculations is a subject of further studies.

For the two individual fields contributing to the high MDR values for the cases T4-03 and T3-06 we calculated field-wise MDR distributions and overlayed them with the delineated CT, as shown in Fig. 6. The high MDR regions intersect remarkably well with the damaged left pre-chiasmatic optic nerve structure, which, for both cases, was associated with the late optic toxicity that was identifiable based on follow-up MR images.

To put the observations in the context to the FLASH effect it should be highlighted that, the dose rates for the clinical cases in this study are well below FLASH dose rates, which are currently considered to be above 40 Gy/s. Furthermore, a FLASH effect is achieved at high dose levels. On the contrary to that, we observe low dose levels delivered at high dose rates. For both index cases the dose delivered at 3.5 Gy/s or more was less than 10% of total dose delivered to the toxicity area. Finally, at this point overwhelming majority of FLASH effect demonstrations are related to acute toxicities, while RION, which is considered in the current study, is late toxicity.

When discussing relative biological effectiveness (RBE), there are numerous parameters that define its characteristics [25]. Synergic effects between dose rate and LET could be explored further and might reveal a better predictive model for late toxicity development. In our investigated cases regions of high LET and regions of high dose rate spatially do not correlate. High dose rates can for example also occur on the proximal edge of a field, where the LET is usually low. If a significant dose-enhancing effect linked to dose rate exists, it is probable that predicting the risk of toxicity would be more accurate by considering the cumulative dose delivered at or above a specific dose rate threshold, rather than solely relying on the instantaneous maximum dose rate. We are further exploring the dose above dose rate parameter space in combination with dose delivered at an elevated RBE due to LET.

The strong limitation of this study is the size of the data set. Fortunately, the incidence of optic apparatus toxicities is low in our patients with intracranial disease [11, 12]. Therefore, the possibility to acquire a sufficiently large data set to prove or disprove the hypothesis will require a multi-institutional collaboration, similar as suggested in the European Particle Therapy Toxicity Workshop (Leuven, Belgium, 04.10.2022). Nevertheless, dose rate calculation for conventional proton treatment plans currently is not a feature readily available in commercial treatment planning systems (TPS). Such calculations require in-house developments or dedicated research builds of TPS.

Eventually, other clinical factors may have influenced the development of the toxicity. For instance, one out of two toxicity cases is a patient more than 60 years old.

In conclusion, our observations reveal large variations in instantaneous dose rates experienced by different volumes within our patient cohort, even when considering the same indications and beam arrangement. Furthermore, we observed an overlap between high-dose rate areas and regions of developed optic toxicities as identified on MR images. At this point, it is not feasible to establish causality between exposure to high dose rates and the development of late optic apparatus toxicities due to the low incidence of injury. Since the number of toxicity cases per institute is generally low, a multi-institutional study would be necessary to provide evidence for the correlation between elevated dose rate and the radiation injury. Further radiobiology studies investigating the effects of low/high dose rate proton exposures will hopefully provide additional insights and further elucidate this potential radiobiological impact.

## Data Availability

No datasets were generated or analysed during the current study.

## Abbreviations

CTCAE: Common Terminology Criteria for Adverse Events
DR: Dose rate
LET: Linear energy transfer
LET_d_: Dose-averaged linear energy transfer
MC: Monte Carlo
MD: Medical doctor
MDR: Voxel-wise maximum dose rate
OAR: Organ at risk
OC: Optic chiasm
ONL: Optic nerve left
ONR: Optic nerve right
RBE: Relative biological effectiveness
RION: Radiation induced optic neuropathy
TPS: Treatment planning system
